# The oral eukaryotic microbiome of *Melanophryniscus admirabilis*, a microendemic and critically endangered toad

**DOI:** 10.7717/peerj.20831

**Published:** 2026-03-24

**Authors:** Cécile Jacry, Michele Bertoni Mann, Michelle Abadie, Márcio Borges-Martins, Jeverson Frazzon, Caroline Isabel Kothe, Ana Paula Guedes Frazzon

**Affiliations:** 1 Independent Researcher, Viry-Châtillon, France; 2 Post-Graduation Program in Agricultural and Environmental Microbiology, Department of Microbiology, Immunology and Parasitology, Universidade Federal do Rio Grande do Sul, Porto Alegre, RS, Brazil; 3 Biosciences Institute, Universidade Federal do Rio Grande do Sul, Porto Alegre, RS, Brazil; 4 Post-Graduation Program in Animal Biology, Department of Zoology, Biosciences Institute, Universidade Federal do Rio Grande do Sul, Porto Alegre, RS, Brazil; 5 Biochemistry and Molecular Biology of Microorganisms Laboratory, Universidade Federal do Rio Grande do Sul, Porto Alegre, RS, Brazil; 6 Institut National de Recherche pour L’agriculture, L’alimentation et L’environnement—INRAE, Institut Micalis, Université Paris-Saclay, Jouy-en-Josas, France; 7 The Novo Nordisk Foundation Center for Biosustainability, Sustainable Food Innovation Group, Kgs., Technical University of Denmark, Lyngby, Denmark

**Keywords:** Oral microbiome, Mycobiome, *Melanophryniscus admirabilis*, Amphibian microbiota, *Bd*-inhibitory fungi, Functional prediction, *18S* rRNA sequencing, Host-microbiome interaction

## Abstract

**Background:**

The oral eukaryotic microbiome of amphibians remains largely unexplored, despite its potential importance for host health and resistance to fungal pathogens such as *Batrachochytrium dendrobatidis* (*Bd*). *Melanophryniscus admirabilis*, a critically endangered red-belly toad species endemic to Brazil and restricted to a 700-m stretch of the Forqueta River, offers unique insights into host-microbiome interactions within highly specialized and threatened neotropical environments. While its narrow distribution limits broader applications, the genus *Melanophryniscus* is widely distributed across South America, potentially serving as a broader model for comparative microbiome research across varied ecological contexts.

**Methods:**

We analyzed the oral eukaryotic microbiota of ten wild *M. admirabilis* using *18S* rRNA gene amplicon sequencing, with the Illumina MiSeq platform. Taxonomic assignments were performed at the phylum, class, and genus levels. Microbial community structure was assessed *via* hierarchical clustering and non-metric multidimensional scaling (NMDS) method based on Bray-Curtis dissimilarity. In addition, functional profiles were inferred from taxonomic data using PICRUSt2 to explore the potential ecological roles of the detected taxa.

**Results:**

Excluding host-derived reads, the predominant fungal phyla identified were Ascomycota and Basidiomycota. Among them, the genus *Malassezia* was present across all samples, suggesting a potentially host-adapted association. Given its known adaptation to mucosal environments and consistent abundance in our dataset, we hypothesize that *Malassezia* may compete with the fungal pathogen *Bd*, potentially acting as a natural microbial protector. Other fungal genera, including *Phlebia*, *Microdochium*, *Fusarium*, and *Rhodotorula*, were detected at lower abundance and may reflect a mixture of commensal, environmental, or opportunistic fungi. Functional prediction analyses revealed signatures of saprotrophic activity and suggested potential metabolic contributions to host-associated niches. The high proportion of unclassified and multi-affiliated sequences highlights the current limitations of reference databases for amphibian-associated eukaryotes, and underscores the value of this study in providing a novel community-level description of oral fungi in a neotropical anuran species.

**Conclusion:**

This study provides the first characterization of the oral eukaryotic microbiome of *M. admirabilis*, revealing a diverse and structured fungal community dominated by *Malassezia*, with predicted functions related to environmental adaptation and fungal competition. These findings suggest that the oral cavity of amphibians harbors functionally active microbial communities that may play a role in pathogen resistance and host-microbe symbiosis.

## Introduction

*Melanophryniscus admirabilis*, commonly known as the admirable red-belly toad, is a microendemic and critically endangered species found exclusively along a 700-m stretch of the Forqueta River, within a fragment of the Atlantic Forest in southern Brazil (Zank et al., 2014). First described in 2006, this species stands out as one of the largest within its genus, with a snout-to-vent length ranging from 29.5 to 40.3 mm (Di-Bernardo, Maneyro & Grillo, 2006).

Due to its highly restricted habitat, *M. admirabilis* is particularly vulnerable to a multitude of anthropogenic disturbances, including hydropower projects, deforestation, illegal pet trade, and the intensive use of pesticides in surrounding agricultural areas. Given its extremely limited distribution and increasing environmental pressures, the species has been classified as Critically Endangered by the International Union for Conservation of Nature (IUCN, 2025). Targeted conservation efforts are essential to ensure the survival of this unique species, which serves as a symbol of the threatened biodiversity within Brazil’s Atlantic Forest (Fonte et al., 2022).

Previous research on *M. admirabilis* has characterized its oral bacterial community, revealing a predominance of Proteobacteria, Firmicutes, Bacteroidetes, and Actinobacteria, and identifying microbial metabolic pathways potentially involved in xenobiotic degradation and adaptation to anthropogenic environments (Mann et al., 2021). In addition, skin cultivable bacteria from *M. admirabilis* were also isolated and demonstrated their contribution to the species’ survival in an environment shaped by anthropic action (Ienes-Lima et al., 2023). While these studies provided valuable insights into the bacterial microbiota, little is known about the eukaryotic community’s composition and potential functional roles inhabiting the oral cavity of *M. admirabilis*.

Eukaryotic microorganisms play significant roles in host health, influencing immunity, metabolism, and interactions within microbial communities. Among them, fungi are particularly relevant, as they can contribute either positively and negatively to the host’s ecological balance. A notable example is *Batrachochytrium dendrobatidis* (*Bd*), a fungus that causes the disease chytridiomycosis in amphibians and has decimated their populations worldwide for decades (Berger et al., 1998; Collins & Storfer, 2003; Kilpatrick, Briggs & Daszak, 2010). This aquatic pathogen spreads through zoospores and infects the keratinized tissues of amphibians, including the mouthparts of tadpoles and the skin of juveniles and adults (Carey et al., 2003; Jani & Briggs, 2014). To date, *Bd* has been detected in approximately 700 amphibian species across multiple continents, and it is currently listed as a significant epidemic by the World Organization for Animal Health due to its severe impact on biodiversity (Carey et al., 2003; Fisher, Garner & Walker, 2009; Schloegel et al., 2010; Scheele et al., 2019). Therefore, a better understanding of the eukaryotic community associated with *M. admirabilis*, with particular attention to fungi, can provide insights into ecological interactions and potential impacts on host health. In this study, we aim to characterize the oral eukaryotic community of *M. admirabilis* using *18S* rRNA sequencing, characterizing the fungal composition, providing novel insights into its ecology and predicting its potential functional roles.

## Materials and Methods

### Samples

Ten oral swab samples were collected from wild *M. admirabilis* individuals (Table S1, sheet ‘metadata’) inhabiting the margins of the Forqueta River in Arvorezinha, Rio Grande do Sul, Brazil, following established amphibian swabbing procedures (Schulte et al., 2011). All fieldwork complied with guidelines from the Chico Mendes Institute for Biodiversity Conservation (ICMBio) and was conducted under SISBIO permits 40004-5 and 10341-1 (issued to M. Borges-Martins). Ethical approval was obtained from the Research and Ethics Committees of the Federal University of Rio Grande do Sul (protocols 19541, 25526, and 25528). To minimize disturbance, each animal was handled for the shortest possible time, a new pair of gloves was used for every individual, and swabbing was performed gently before immediately releasing the toad at its capture site. These procedures are integrated into a long-term monitoring and conservation program for the only known population of the admirable red-belly toad and follow the guidelines outlined in the Action Plan for the Conservation of Amphibians and Reptiles in Southern Brazil.

### DNA extraction and 18S sequencing

Total genomic DNA was extracted from each oral swab using the DNeasy Blood & Tissue Kit (Qiagen, Hilden, Germany), following the manufacturer’s protocol, as commonly applied in amphibian microbiome studies (*e.g*., Schulte et al., 2011). DNA quantity was measured with a Qubit fluorometer (Thermo Fisher Scientific, Waltham, MA, USA), and DNA purity was assessed using a NanoDrop ND-1000 spectrophotometer. The samples were amplified using the primers nu-SSU-0817-5′ (TTAGCATGGAATAATRRAATAGGA) and nu-SSU-1196-3′ (TCTGGACCTGGTGAGTTTCC), targeting the V4 (partial) and V5 variable regions of the *18S* rRNA gene, with Illumina adapter sequences added. These primers, referred to as R1 and R2 in subsequent sections of the study, were originally described by Borneman & Hartin (2000) and were selected for their ability to amplify a conserved region of fungal *18S* rRNA, allowing broad detection of fungal taxa. Amplification parameters were adapted from the original protocol, with minor modifications optimized for *18S* rRNA sequencing on the Illumina MiSeq platform. PCR conditions were as follows: initial denaturation at 95 °C for 3 min; 30 cycles of 98 °C for 45 s, 56 °C for 50 s, and 72 °C for 90 s; followed by a final extension at 72 °C for 5 min. PCR products were purified using Agencourt AMPure XP beads (Beckman Coulter, Indianapolis, IN, USA) according to the manufacturer’s protocol. Indexes were then added to the DNA libraries following the instructions provided by the manufacturer (Illumina Page 5/15 Inc., San Diego, CA, USA). Sequencing of the libraries was performed on the Illumina MiSeq platform using the MiSeq Reagent Kit v2 (500 cycles).

### Eukaryotic and fungal community analysis

The quality of raw sequencing data was assessed using FastQC v.0.12.1 (Andrews, 2010). Paired-end reads generated from R1 and R2 primers were merged with PEAR v.0.9.6.4, with default parameters (Zhang et al., 2014) and processed in FROGS v.4.1.0 (Escudié et al., 2018; Bernard et al., 2021) to generate Amplicon Sequence Variants (ASVs). Clustering was performed using SWARM v.3.2 (Mahé et al., 2021) with an aggregation distance of 1. Chimeras and low-abundance ASVs (<0.01% of total reads) were removed, and the remaining sequences were taxonomically assigned using the PR2 v.5.0.1 *18S* rRNA reference database (Guillou et al., 2013).

Each fungal class identified through PR2, we conducted a targeted review of the scientific literature and searched for evidence of (i) direct *Bd* growth inhibition or (ii) production of antifungal secondary metabolites by any member of that class. The outcome of this assessment is presented in Table 1, which lists the fungal classes detected along with their documented *Bd*-inhibitory potential, supported by the corresponding references when applicable.

**Table 1:** *Bd*-inhibitory fungal classes detected in buccal samples of *M. admirabilis*. The table lists fungal classes identified in the dataset that are known or suspected to include species with antifungal activity against *Bd*, the pathogen responsible for chytridiomycosis in amphibians. Evidence is based on published literature describing either direct inhibition of *Bd* or the production of antifungal secondary metabolites.

Fungal class	Related phylum	*Bd* inhibition potential	Explanation	References
Dothideomycetes	Ascomycota	Yes	Contains genera like *Cladosporium;* known to have *Bd*-inhibitory activity; some strains have been known to produce antifungal secondary metabolites.	Loudon et al. (2014), Kearns et al. (2017)
Eurotiomycetes	Ascomycota	Yes	Includes *Penicillium* and *Aspergillus*, shown *Bd-*inhibitory properties and known to produce antifungal protein (AFPs).	Kearns et al. (2017), Garrigues et al. (2018)
Saccharomycetes	Ascomycota	Yes	Includes *Debaryomyces* and *Candida*; some strains have shown antifungal activity against *Bd*.	Kearns et al. (2017)
Sordariomycetes	Ascomycota	Yes	Contains genera like *Fusarium*, *Chaetomiaceae* and *Pestalotiopsis*; some strains have been known to produce antifungal secondary metabolites and shown antifungal activity against *Bd*.	Loudon et al. (2014), Kearns et al. (2017)
Agaricomycetes	Basidiomycota	Yes	Includes saprophytic forest fungi; known to have *Bd*-inhibitory activity.	Kearns et al. (2017)
Tremellomycetes	Basidiomycota	Yes	Includes *Cryptococcus* and *Trichosporon*, some strains of which produce antifungal compounds.	Kearns et al. (2017)
Leotiomycetes	Ascomycota	Yes	Includes phytopathogenic and saprophytic fungi; *Bd-*inhibition observed in *Neobulgaria* and *Pseudeurotium*.	Alexiev et al. (2023)
Exobasidiomycetes	Basidiomycota	Possible	Mostly plant parasites; includes *Tilletiopsis* species producing antifungal compounds.	Urquhart & Punja (2002)
Microbotryomycetes	Basidiomycota	Possible	Contains some yeast-like fungi; genera like *Rhodotorula* can inhibit or enhance *Bd* growth.	Kearns et al. (2017)
Wallemiomycetes	Basidiomycota	No	Environmental extremophiles; no evidence of *Bd* interaction.	N/A.
Cystobasidiomycetes	Basidiomycota	No	Basidiomycetous yeasts; no evidence of antifungal activities.	N/A.
Lecanoromycetes	Ascomycota	No	Mostly lichen-forming fungi; Known to produce secondary metabolites with antifungal activity, though no specific data on *Bd-*inhibition.	Zambare & Christopher (2012), Furmanek, Czarnota & Seaward (2019)
Arthoniomycetes	Ascomycota	No	Also lichenized or plant-associated; no evidence of *Bd* interaction.	N/A.
Chytridiomycetes	Chytridiomycota	Contains *Bd*	Includes *Batrachochytrium dendrobatidis* itself, the amphibian pathogen of concern.	Longcore, Pessier & Nichols (1999), Berger et al. (2005), Fisher et al. (2009)

**Note:**

*Bd*, *Batrachochytrium dendrobatidis*; N/A, not available.

All downstream analyses were carried out in RStudio v.3.6.1 using the *phyloseq*, *ggplot2*, *vegan*, *dendextend*, and *cluster* packages (McMurdie & Holmes, 2013; Galili, 2015; Wickham, 2016; Maechler et al., 2022; Oksanen et al., 2022). To account for sequencing-depth variation, ASV counts were converted to relative abundances before beta-diversity analyses. Bray-Curtis dissimilarities were computed on these normalized data.

Community structure was visualized using non-metric multidimensional scaling (NMDS) computed with *metaMDS* function from the *vegan* package. A fixed random seed was set prior to the analysis (set.seed(42)) to ensure full reproducibility, as the NMDS algorithm begins from a random initial configuration of samples in multidimensional space. The ordination was performed in two dimensions (*k* = 2) with 100 iterations (trymax = 100) to ensure convergence. The quality of the ordination was assessed using the stress value, which quantifies the mismatch between the observed dissimilarities and their representation in the reduced-dimensional space. A stress-based evaluation was used to verify that the two-dimensional solution provided an adequate representation of the underlying distance structure, following conventional thresholds for ecological community data (*e.g*., stress <0.1 generally indicating a reliable ordination).

Hierarchical clustering was performed using the Unweighted Pair Group Method with Arithmetic Mean (UPGMA) *via* the *hclust* function in R with method = “average” applied to the same Bray-Curtis distance matrix. The optimal number of clusters (k = 2–10 tested) was determined using *silhouette width*, selecting the k that maximized mean silhouette value. A complete silhouette plot was generated to evaluate cluster separation. The final dendrogram was visualized and enhanced with *dendextend*, with branches colored by cluster membership and labels colored by host sex to aid biological interpretation.

### Functional predictions from 18S sequences

The functional potential of the eukaryotic microbiota in *M. admirabilis* was predicted using PICRUSt2 v.4.1.0 (Douglas et al., 2020). Sequences were processed through three main steps: phylogenetic placement, functional abundance and pathway abundance estimations, implemented using the FROGS pipeline (Bernard et al., 2021). The predicted functions were classified using the EC (Enzyme Commission) database mapping table, with MetaCyc as the reference for pathway annotation (Caspi et al., 2014).

## Results

### Taxonomic composition

A total of 666,709 assembled reads were obtained from *18S* rRNA sequencing of ten buccal samples collected from wild *M. admirabilis*, following merging with the PEAR tool (Table S1, sheet ‘PEAR’). After clustering and quality filtering, 129 ASVs were retained for downstream taxonomic analysis (Table S1, sheet ‘18S_PR2’). Rarefaction curves (Fig. S1) indicated that sequencing depth was sufficient to capture the microbial diversity present in each sample. Samples TA07, TA11, and TA12 displayed the highest species richness, while TA05 showed the lowest.

**Figure 1:**
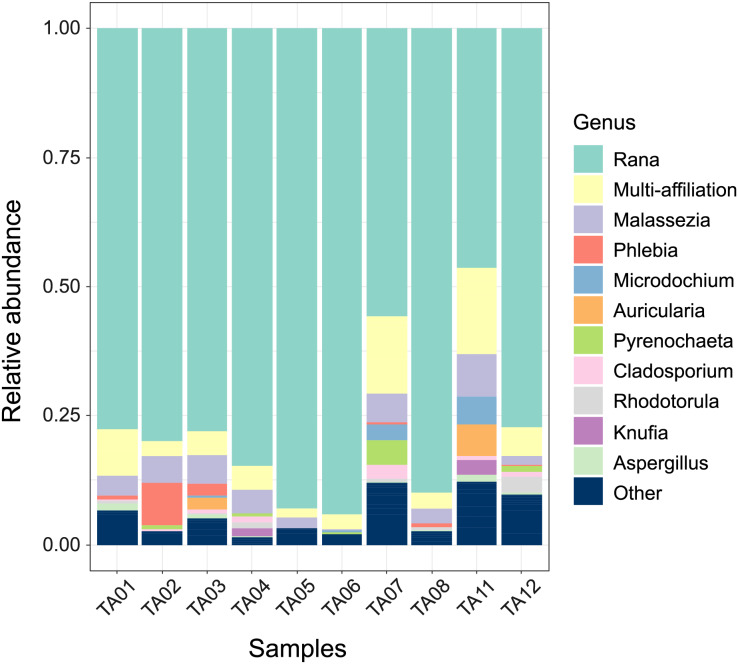
Taxonomic profile of the oral eukaryotic community of *M. admirabilis* at the genus level. Relative abundances of detected genera across the ten oral samples, with each bar representing an individual. Colors indicate the proportional contribution of each genus within each sample. The category “Other” includes all genera that do not belong to the 11 most abundant genera, based on their cumulative relative abundance across all samples.

To characterize the eukaryotic community, we analyzed taxonomic diversity at the phylum, class (Fig. S2), and genus levels (Fig. 1). A total of 11 phyla, 27 classes, and 63 genera were identified across all samples (Table S1, sheets ‘phylum’, ‘class’, ‘genus’).

At the phylum level (Fig. S2A), Chordata was the dominant group across all samples (77.99%, Table S1, sheet ‘phylum’), most likely reflecting the contribution of host-derived sequences. Among fungal phyla, Ascomycota (10.46%) and Basidiomycota (10.23%) were the most abundant in our samples. A small proportion of reads was assigned to Chytridiomycota (0.17%), a phylum that includes *Bd*. Reads assigned to Arthropoda represented 0.38% of the dataset, a level consistent with incidental detection related to the insectivorous diet of amphibians. Several other taxa were recovered at similarly low abundances. For example, Ciliophora (0.51%) represent protist taxa, while Apicomplexa (0.06%) and Platyhelminthes (0.01%) are recognized as common parasites of vertebrate mucosa. Very low proportions of Gyrista (0.12%), Cercozoa (0.03%), and certain Opisthokonta (0.05%) were also detected, most likely reflecting environmental contamination or indirect associations rather than true colonization.

Given that fungi were the most frequently observed eukaryotic microorganisms in our dataset (Fig. S2B), we focused our analysis on this kingdom and its potential interactions with *M. admirabilis*. A total of 14 fungal classes were detected, representing a variety of ecological strategies including saprotrophs, symbionts, pathogens, and extremophiles. Among these, members of Basidiomycota, such as Agaricomycetes (4.70%), Exobasidiomycetes (4.01%), Tremellomycetes (0.87%), Cystobasidiomycetes (0.48%), and Microbotryomycetes (0.14%). Ascomycota were also well represented, notably Dothideomycetes (4.48%), Sordariomycetes (2.94%), Eurotiomycetes (1.45%), and Saccharomycetes (0.89%) (Table S1, sheet ‘class’). Based on a literature review of documented *Bd*-inhibitory taxa, these classes were categorized according to their potential antifungal properties (Table 1).

At the genus level, *Malassezia* emerged as the most consistently detected fungal taxon across oral samples, with an average relative abundance of 4.01% (Fig. 1, Table S1, sheet ‘genus’). Although other groups such as *Rana* (Chordata) and “multi-affiliated” sequences were more abundant overall, *Malassezia* represented the most prominent identified fungal genus. Its dominance, however, was not uniform across all samples (*e.g*. in TA02 *Phlebia* (8.28%) exceeded *Malassezia* (5.27%) in relative abundance).

Sequences classified as “multi-affiliated” accounted for 6.52% of total reads, reflecting cases where sequences matched multiple taxa with similar scores. By contrast, sequences labeled as “unknown” could not be confidently assigned to any taxon in the reference database. For the purposes of this study, both categories were considered ambiguous taxonomic assignments, underscoring the limitations of current reference databases for amphibian-associated eukaryotes.

Several other fungal genera were detected at relatively low abundances and showed variable distributions among individuals, suggesting localized or transient colonization. For instance, *Phlebia* (1.27%) was found in higher abundance in TA02 and TA03, but at much lower levels in other samples. *Microdochium* (0.92%) was predominantly present in TA07 and TA11, further supporting the idea of sample-specific colonization by opportunistic or saprotrophic fungi. *Auricularia* (0.84%) was detected exclusively in TA03 and TA11, while *Pyrenochaeta* (0.78%) was mainly observed in TA07 and TA12, indicating potential host- or environment-specific occurrences. Additionally, several other less abundant genera, including *Cladosporium* (0.72%), *Rhodotorula* (0.59%), *Aspergillus* (0.44%), *Cryptococcus* (0.42%), *Penicillium* (0.35%), and *Fusarium* (0.29%), were detected at varying abundances across samples, reflecting both the taxonomic diversity and sporadic distribution of fungal taxa colonizing the amphibian oral cavity.

**Figure 2:**
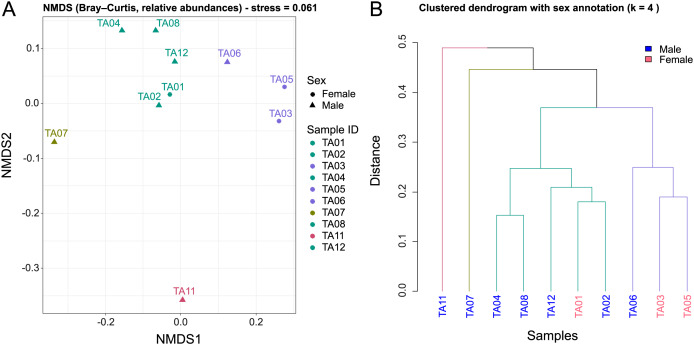
Beta-diversity patterns of the oral eukaryotic microbiota of *M. admirabilis*. (A) Non-metric multidimensional scaling (NMDS) ordination based on Bray-Curtis dissimilarities computed from relative abundance data. Each point represents an individual buccal sample. Colors indicate the grouping structure observed among samples, while sex is indicated by point shape (● = female, ▲ = male). (B) Hierarchical clustering dendrogram constructed using the same Bray-Curtis distance matrix. Branch colors represent the optimal number of clusters identified by silhouette analysis. Sample labels are colored according to sex (blue = male, pink = female).

To assess the structure of fungal communities among individuals, a non-metric multidimensional scaling (NMDS) analysis based on Bray-Curtis dissimilarity was performed, followed by hierarchical clustering (Fig. 2). This combined approach enabled comparison of community composition across individuals and detection of potential ecological or biological patterns.

The NMDS ordination (Fig. 2A) revealed distinct patterns in community composition (stress = 0.061), indicating that the two-dimensional representation accurately reflects the ecological distances between individuals. Samples positioned near each other exhibited comparable taxonomic structures, while those farther apart differed substantially in composition. Several samples clustered closely together in the ordination space (*e.g*., TA04-TA08-TA12; TA01-TA02), suggesting similar oral eukaryotic communities, whereas TA07 and TA11 appeared as clear outliers with markedly divergent profiles. No consistent structuring by sex was detected; however, the unbalanced sex ratio (7:3) and limited sample size preclude strong conclusions.

Hierarchical clustering was performed using the Unweighted Pair Group Method with Arithmetic Mean (UPGMA). The resulting dendrogram reflects relative dissimilarities between samples, with shorter branch lengths indicating greater similarity. The optimal number of clusters (k = 4) was determined using the silhouette method, which assesses how well each sample fits within its assigned cluster compared to neighboring ones (Fig. S3A). Clustering quality was further evaluated using silhouette plots, which quantify the consistency of each sample’s classification. Silhouette values range from −1 (poor clustering) to 1 (optimal clustering), allowing identification of individuals weakly associated with their assigned group. Samples with low silhouette values (≈0) may represent intermediates between clusters, or constitute true outliers (Fig. S3B). Silhouette analysis showed that most samples were well assigned to their respective clusters, with silhouette widths above 0.3. However, two samples (TA07 and TA11) displayed notably low silhouette scores (≈0; Table S1, sheet ‘silhouette_widths’), indicating ambiguous cluster membership. These samples exhibited similar dissimilarities to their assigned cluster and to neighboring clusters, suggesting transitional or distinct microbial community profiles.

Dendrogram branches were color-coded according to the identified clusters, and leaf labels were annotated by individual sex to explore potential biological structuring (Fig. 2B). The dendrogram supported the groupings observed in the NMDS ordination, revealing four distinct community structures: two main clusters (Cluster 1: TA04, TA08, TA12, TA01, and TA02; Cluster 2: TA06, TA03, and TA05) and two outlier profiles (TA07 and TA11).

The concordance between the ordination and clustering analyses reinforces the robustness of the identified groupings. Collectively, these results reveal four distinct oral eukaryotic community structures in *M. admirabilis*, with TA07 and TA11 exhibiting particularly divergent profiles. Overall, these findings highlight substantial intra-population variability in the oral eukaryotic microbiota of this species.

### Functional prediction analysis

**Figure 3:**
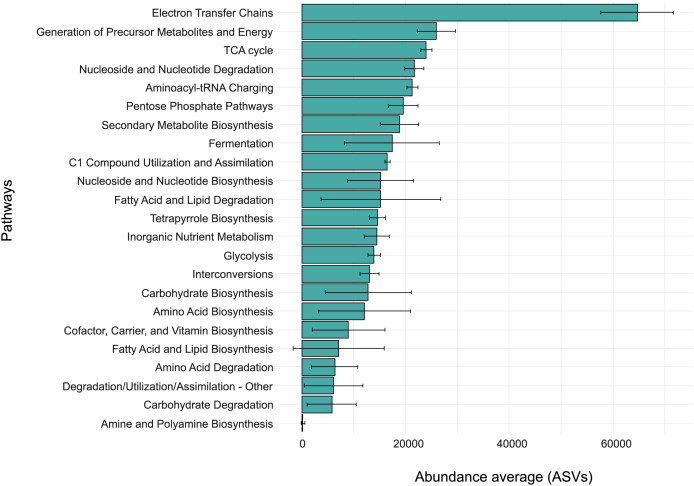
Predicted metabolic functions of the oral eukaryotic microbiome of *M. admirabilis*. Distribution of metabolic pathway categories predicted from the level 2 classification of the MetaCyc database, based on *18S* rRNA gene profiles from ten oral samples of wild *M. admirabilis*. The x-axis represents the average abundance (ASVs), while the y-axis lists the predicted metabolic functions.

To explore the potential metabolic capabilities of the oral eukaryotic microbiome of *M. admirabilis*, we performed functional predictions using PICRUSt2 based on the taxonomic composition. The predicted functions were grouped into major metabolic categories (Fig. 3; Table S1, sheets ‘FROGSFUNC’ and ‘FROGSFUNC_Average’).

Among all categories, “*Electron Transfer Chains*” exhibited the highest relative abundance, suggesting a community dominated by aerobic eukaryotes relying on oxidative phosphorylation. Other energy-related pathways, including “*TCA Cycle*” and “*Generation of Precursor Metabolites and Energy*,” were also enriched, indicating a robust core metabolism. Moreover, the low variability observed in these core pathways across samples indicates their conserved and fundamental role within the oral eukaryotic microbiota.

Several biosynthetic and catabolic pathways also displayed moderate relative abundances. Notably, “*Aminoacyl-tRNA Charging*”, the “*Pentose Phosphate Pathway*”, and “*Secondary Metabolite Biosynthesis*” were well represented, reflecting essential cellular functions. The prominent abundance of the “*Nucleoside and Nucleotide Degradation*” pathway may indicate active nucleic acid recycling. The presence of “*Fermentation*” and “*Glycolysis*” pathways suggests a capacity for fermentative metabolism. Additionally, “*Fatty Acid and Lipid Degradation*” and “*Fatty Acid and Lipid Biosynthesis*” were relatively enriched, though these pathways showed marked interindividual variability. In contrast, pathways such as “*Amine and Polyamine Biosynthesis*”, “*Carbohydrate Degradation*”, and “*Degradation/Utilization/Assimilation-Other*” were poorly represented.

At the 4th level of functional annotation, we observed the presence of ecologically relevant pathways including “*octane oxidation*”, “*chitin degradation*”, and the “*mevalonate pathway I*” (Table S1, sheets ‘FROGSFUNC’ and ‘FROGSFUNC_Average’).

## Discussion

In this study, we characterized the oral eukaryotic community of *M. admirabilis* using *18S* rRNA sequencing. Our results revealed that fungi communities were dominated by members of Ascomycota and Basidiomycota, mainly represented by environmental saprotrophic taxa known to participate in the decomposition of plant material. Their presence in the toad’s oral cavity likely reflects habitat exposure, but may also point to symbiotic associations with yeasts such as *Debaryomyces* and *Cryptococcus*, both identified in our dataset (Castelo-Branco et al., 2021).

The variability in fungal phylum abundance also likely results from environmental exposure and host-microbiota dynamics. Many of these fungi, including those in Ascomycota and Basidiomycota, are involved in decomposition or symbiosis (Castelo-Branco et al., 2021; Zambrano-Forero et al., 2023) and may possess antifungal properties effective against *Bd* (Kearns et al., 2017). Although *Bd* was not detected, sequences from Chytridiomycota, a phylum including *Bd*, were present at low abundance, suggesting possible environmental contamination or early colonization stages.

Beyond fungi, genuine protists such as Ciliophora were detected, along with arthropod-derived sequences that reflect the toad’s diet. As *M. admirabilis* feeds exclusively on arthropods, including Formicidae, Acari, and Coleoptera (Lima, 2014), the presence of arthropod DNA is consistent with trophic intake and ecological specialization (Duellman & Trueb, 1994; Wells, 2010).

Given the prevalence and diversity of fungi, we focused on their taxonomic roles and potential functional relevance (Table 1). Several detected fungal classes (*e.g*., Dothideomycetes, Sordariomycetes, Eurotiomycetes) contain genera such as *Aspergillus*, *Penicillium*, *Fusarium*, and *Candida*, which have been reported to produce antifungal compounds or to inhibit *Bd* (Loudon et al., 2014; Kearns et al., 2017).

Among these fungi, *Malassezia* was the most consistently detected genus across individuals. This lipophilic yeast, common on the skin and in the oral microbiomes of vertebrates, including amphibians (Kobayashi, 2008; Spatz & Richard, 2020), may be well adapted to the moist, lipid-rich oral mucosa of *M. admirabilis*. Amphibian anurans possess adhesive tongues covered with a mucus layer and characterized by a surface rich in fungiform and filiform papillae (Kleinteich & Gorb, 2016, 2017). In bufonids, the non-keratinized and highly flexible tongue epithelium remains continuously moist, creating a stable humid microenvironment.

These anatomical and biochemical features likely generate a lipid-modified niche that can support the persistence of lipophilic yeasts such as *Malassezia*. We hypothesize that this may be further enhanced by the surfactant-like properties of amphibian saliva, which can emulsify lipid compounds into micelles or fine droplets, increasing their bioavailability for lipid-dependent fungi. When combined with potentially low antimicrobial pressure and stable microclimatic conditions within the oral cavity, these factors likely contribute to the establishment and maintenance of *Malassezia* in *M. admirabilis*.

Notably, *Malassezia* may also compete with *Bd* for this niche, acting as a potential microbial barrier to *Bd* colonization. Our findings align with those of Ienes-Lima et al. (2023), who reported no *Bd* presence in *M. admirabilis* skin using nested PCR, reinforcing the idea that *Bd* is not a resident of this species’ mycobiome. The observed interindividual variability in fungal composition likely reflects environmental exposure, diet, and host-specific factors (Pontes et al., 2025).

Functional profiling indicates that the oral community displays metabolic processes. Pathways related to energy production, particularly oxidative phosphorylation (*e.g*., TCA cycle, electron transport), were strongly represented, suggesting a dominance of aerobic, active eukaryotes such as protists and fungi. Moderate enrichment in biosynthetic pathways (*e.g*., aminoacyl-tRNA charging, secondary metabolite biosynthesis) supports this interpretation.

The presence of fermentation and glycolysis pathways suggests that certain taxa may adapt to microaerophilic conditions within the oral cavity, perhaps shaped by mucus composition, feeding activity, or host physiology. High variability in lipid metabolism across samples suggests interindividual differences in ecological or physiological conditions.

Intriguingly, pathways such as chitin degradation and octane oxidation, linked to insect exoskeleton digestion and pollutant breakdown, respectively, suggest potential functional roles aligned with the host’s ecology. The detection of the mevalonate pathway I, associated with antimicrobial compound biosynthesis, also suggests a possible role in microbial defense, echoing similar bacterial findings in *M. admirabilis* (Mann et al., 2021).

These findings point to a potential role of the oral mycobiome in limiting pathogen colonization, particularly in contexts where human activity increases pathogen exposure. These results establish the first detailed taxonomic and functional profile of the oral eukaryotic community in *M. admirabilis*, thereby providing an essential baseline to guide future investigations into microbial interactions and to explore the potential contributions of oral eukaryotes to host-microbe dynamics in amphibians. Furthermore, the high proportion of sequences classified as ‘unknown’ or ‘multi-affiliated’ underscores major gaps in current reference databases for amphibian-associated fungi. While these gaps limit the completeness of taxonomic resolution, they also highlight how little is known about these communities. In this context, our study provides ecologically valuable baseline data by offering the first detailed description of the oral mycobiome of a critically endangered species and identifying fungal taxa that may contribute to pathogen resistance.

## Conclusions

This study presents the first comprehensive characterization of the oral eukaryotic microbiome in *M. admirabilis*, revealing a diverse and structured fungal community. Among the identified taxa, *Malassezia* was consistently detected across all oral samples and, on average, emerged as the most abundant genus, although dominance varied between individuals. Functional predictions further indicate that the oral eukaryotic community may possess metabolic capacities relevant to nutrient processing, microbial competition, and potential pathogen suppression.

Our findings offer preliminary insight into the conditions that may favor the persistence of *Malassezia* in this environment. The potential interplay between the yeast’s lipophilic metabolism and the surfactant-like properties of amphibian saliva points toward a host-mediated mechanism facilitating fungal colonization. This observation raises several important questions: (i) Which biochemical components of amphibian saliva promote the persistence of lipophilic yeasts? (ii) How strongly does host physiology influence the assembly of oral fungal communities in amphibians? (iii) Is the association with Malassezia a species-specific feature or part of a broader pattern among anurans?

We further note that addressing these questions will require characterizing the biochemical composition of *M. admirabilis* saliva, identifying lipid metabolites in the oral cavity, and applying targeted metagenomics to refine fungal community profiling. Isolating *Malassezia* strains and assessing their lipolytic capacities would also help test the hypothesized functional link.

In addition, the consistent detection of *Malassezia* raises broader questions about its potential ecological interactions with the amphibian pathogen *Bd*. While direct inhibition of *Bd* by *Malassezia* has not been demonstrated, its persistence in the oral mucosa and potential competition for nutrients or physical space may influence opportunities for *Bd* colonization. This opens further avenues for investigating whether oral fungal communities contribute to natural variation in *Bd* susceptibility among amphibian species, and through which ecological mechanisms (*e.g*., resource competition, niche exclusion, or metabolite-mediated interactions) such effects may occur.

As the first report of the oral fungal community in a wild population of *M. admirabilis*, this study expands the current understanding of amphibian microbiomes and contributes to the ecological knowledge of a critically endangered Neotropical species. Further exploration of both fungal and bacterial members of the oral microbiome, along with their functional roles, will be crucial for improving reference databases and identifying potential symbiotic or antagonistic relationships. Ultimately, this work provides new perspectives on the ecology of amphibian-associated fungal communities by documenting, for the first time, the taxonomic composition and predicted functional potential of the oral eukaryotic microbiome in a microendemic toad. While the taxa identified are not novel, our findings extend the taxonomic and ecological baseline for amphibian mycobiomes and underscore the importance of the oral cavity as an overlooked but potentially informative niche for host-microbe interaction studies in threatened amphibians.

## Supplemental Information

10.7717/peerj.20831/supp-1 Supplemental Information 1 Supplemental Tables – Oral microbiota of *Melanophryniscus admirabilis*. Supporting data related to the oral eukaryotic microbiome of *Melanophryniscus admirabilis*:
Sheet “metadata”: Metadata of the ten oral swab samples collected from wild *M. admirabilis*.
Sheet “PEAR”: Statistical summary of pair-end read assembly for each sample using the PEAR software.
Sheet “18_PR2”: Taxonomic assignment of filtered ASVs using the PR2 v5.0.1 18S rRNA reference database.
Sheet “phylum”: Relative abundance and average percentages of eukaryotic phyla detected in the oral samples.
Sheet “class”: Relative abundance and average percentages of eukaryotic classes in the oral samples.
Sheet “genus”: Relative abundance and average percentages of eukaryotic genera detected in the oral samples.
Sheet “silhouette_widths”: Sample-specific silhouette widths derived from Bray–Curtis dissimilarity, used to assess clustering performance.
Sheet “FROGFUNC”: Predicted functional profiles of the oral eukaryotic microbiome using PICRUSt2 and the MetaCyc database.
Sheet “FROGFUNC_Average”: Summary of functional pathway abundances and variability across samples.

10.7717/peerj.20831/supp-2 Supplemental Information 2 Rarefaction curves of microbial diversity across different samples. Rarefaction curves illustrate the relationship between sequencing depth and observed microbial diversity in ten different samples. The x-axis represents the number of sequences (or reads), while the y-axis indicates the cumulative number of observed species.

10.7717/peerj.20831/supp-3 Supplemental Information 3 Taxonomic profile of the oral eukaryotic community of *M. admirabilis* at higher taxonomic ranks. Relative abundances of detected taxa at the phylum (A) and class (B) levels across the ten oral samples, with each bar representing an individual. Colors indicate the proportional contribution of each taxon within each sample. In panel (B), the category “Other” includes all taxa that do not belong to the 11 most abundant classes, based on their cumulative relative abundance across all samples.

10.7717/peerj.20831/supp-4 Supplemental Information 4 Optimal number of clusters determined by silhouette analysis. (A) Average silhouette width for k = 2 to 10 clusters. The maximum average silhouette width indicates that k = 4 is the optimal number of clusters. (B) Cluster membership of individual samples for k = 4. Each horizontal bar represents one sample, colored according to its assigned cluster (Cluster 1 = red; Cluster 2 = green). Samples in the same cluster are grouped together for visual clarity.

## Additional Information and Declarations

### Data Availability

The following information was supplied regarding data availability:

The raw sequence data are available at NCBI BioProject: PRJNA1218443.
