# Supplementary figures and images for: The oral eukaryotic microbiome of Melanophryniscus admirabilis, a microendemic and critically endangered toad
Source: PeerJ. 2026 Mar 24;14:e20831. doi: 10.7717/peerj.20831 (PMC13024235; doi:10.7717/peerj.20831)

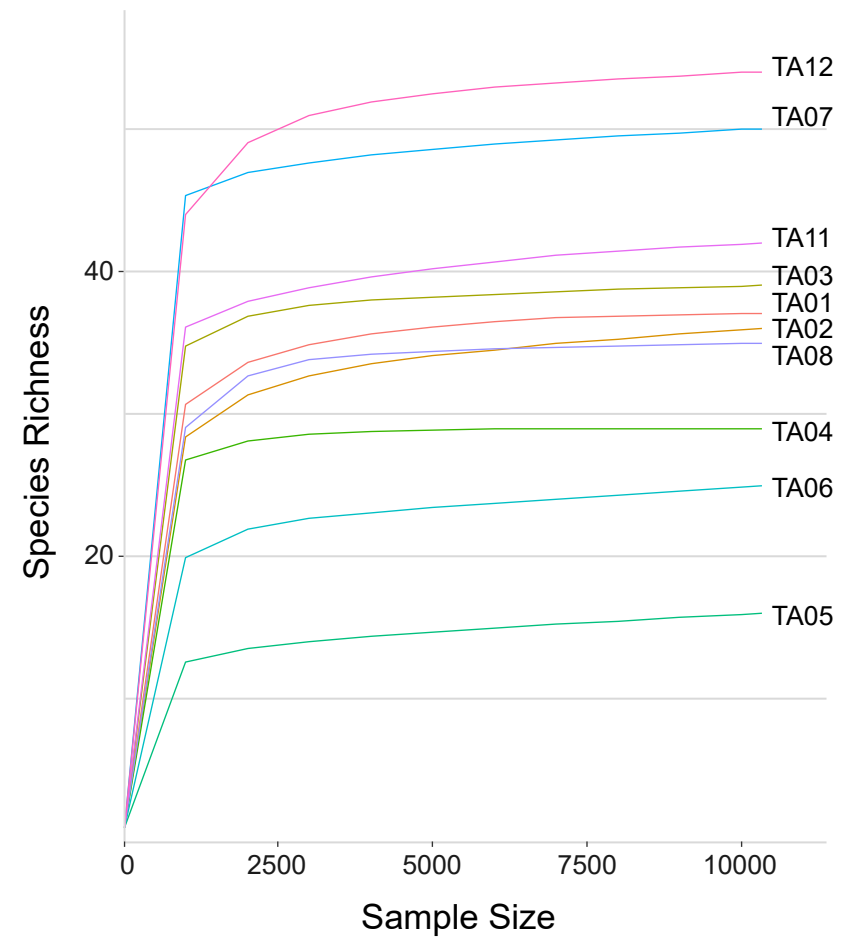

Supplement: Supplemental Information 2 — Rarefaction curves illustrate the relationship between sequencing depth and observed microbial diversity in ten different samples. The x-axis represents the number of sequences (or reads), while the y-axis indicates the cumulative number of observed species. [file peerj-14-20831-s002.pdf]

A

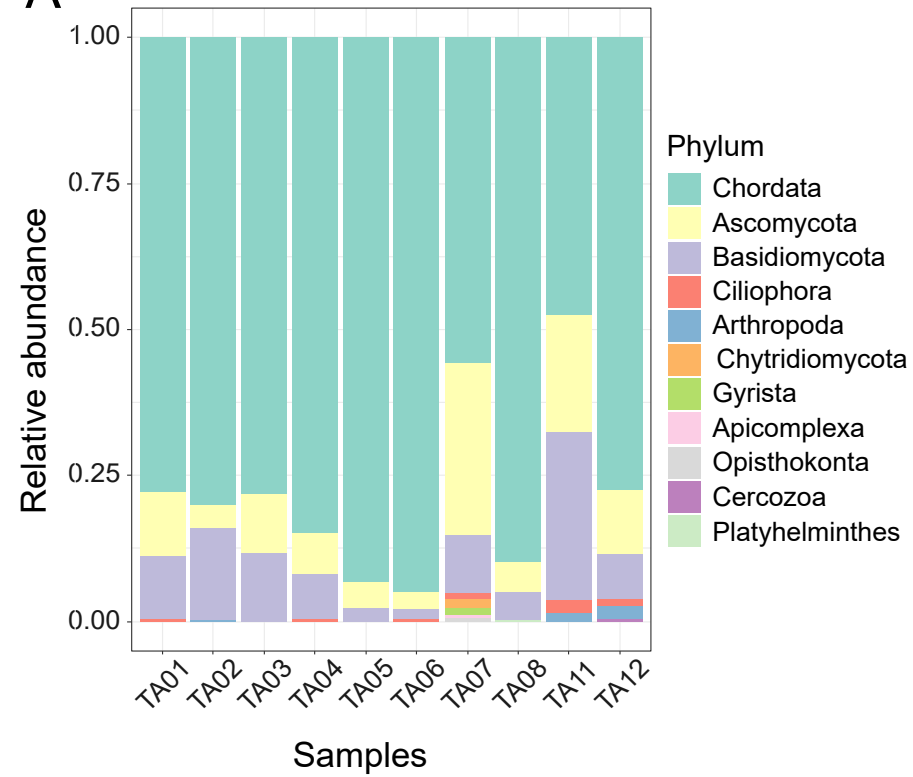

B

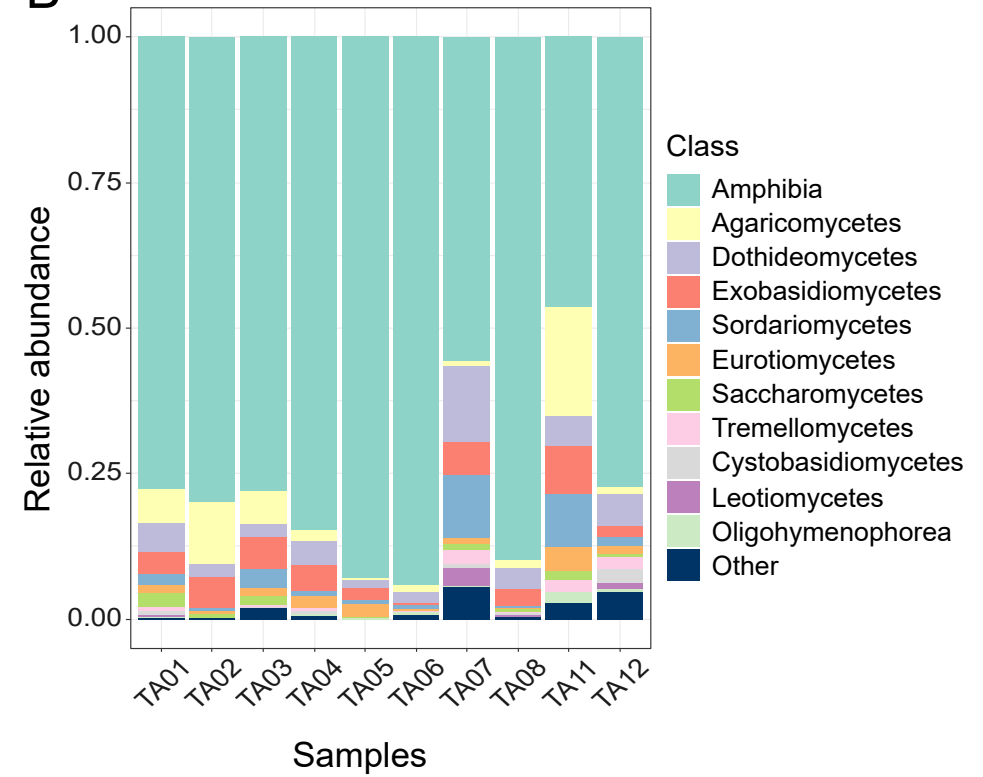

Supplement: Supplemental Information 3 — Relative abundances of detected taxa at the phylum (A) and class (B) levels across the ten oral samples, with each bar representing an individual. Colors indicate the proportional contribution of each taxon within each sample. In panel (B), the category “Other” includes all taxa that do not belong to the 11 most abundant classes, based on their cumulative relative abundance across all samples. [file peerj-14-20831-s003.pdf]

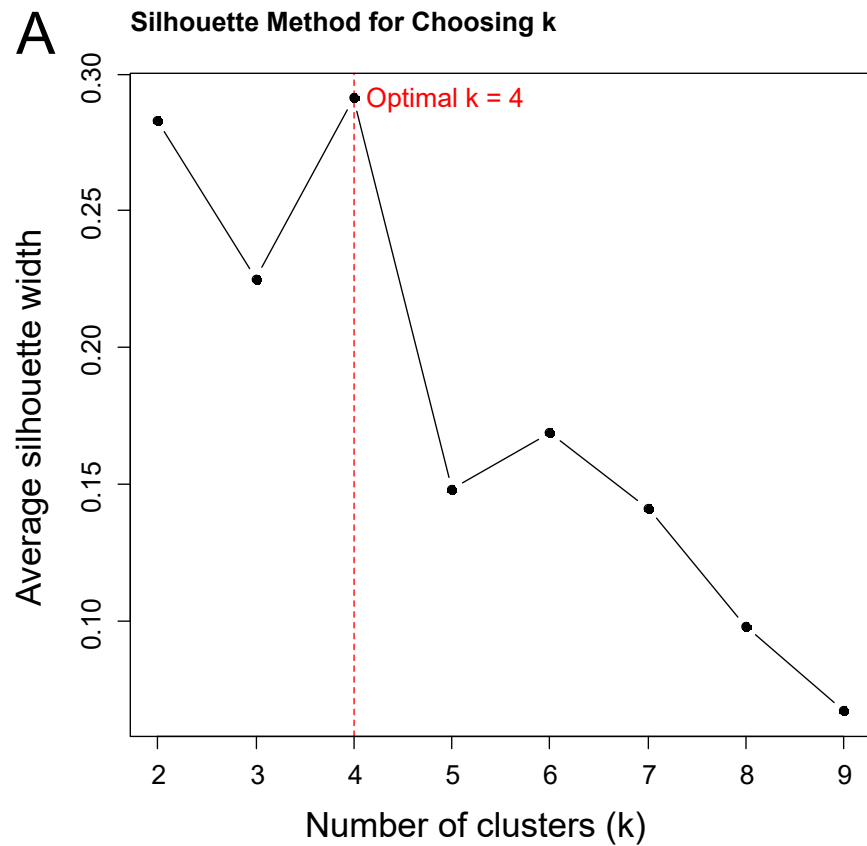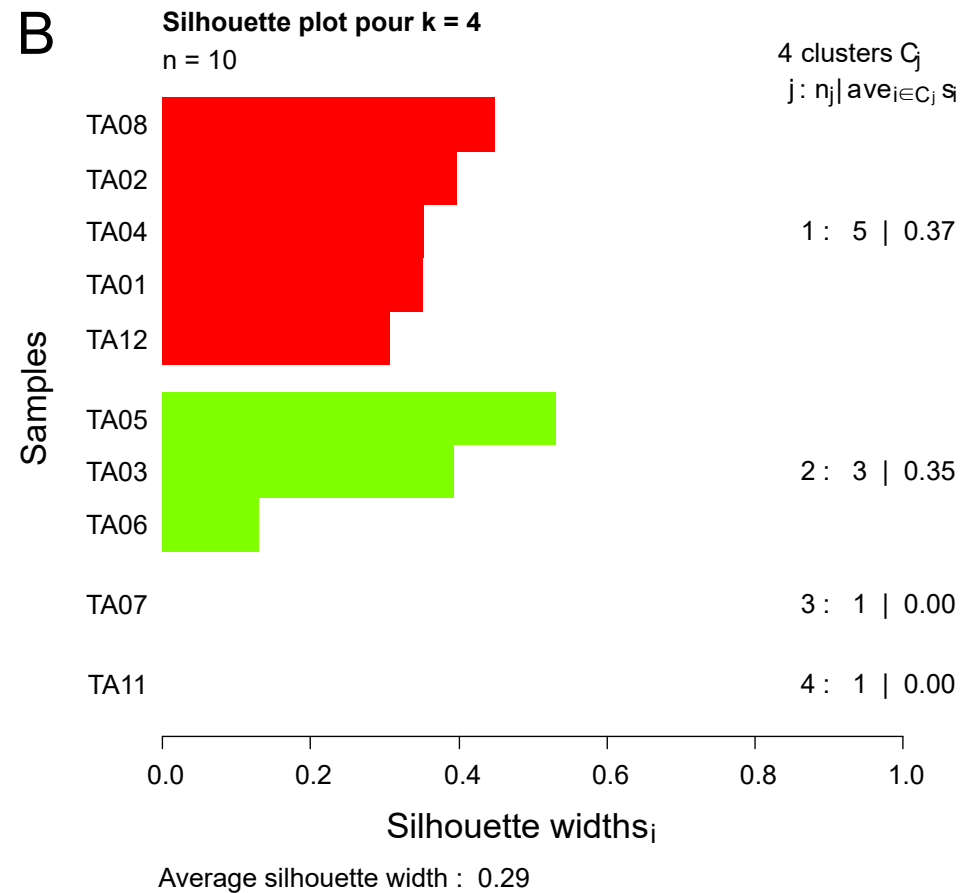

Supplement: Supplemental Information 4 — (A) Average silhouette width for k = 2 to 10 clusters. The maximum average silhouette width indicates that k = 4 is the optimal number of clusters. (B) Cluster membership of individual samples for k = 4. Each horizontal bar represents one sample, colored according to its assigned cluster (Cluster 1 = red; Cluster 2 = green). Samples in the same cluster are grouped together for visual clarity. [file peerj-14-20831-s004.pdf]
